# Analysis of microRNA Expression after Glutamine Intervention in Acute Renal Ischemia-Reperfusion Injury

**DOI:** 10.1155/2022/2401152

**Published:** 2022-01-05

**Authors:** Suhua Li, Xuan Huang, Shun Wang, Xueqian Chu, Munire Aierken

**Affiliations:** Nephrology Center, The First Affiliated Hospital of Xinjiang Medical University, Xinshi District, Urumqi 830054, China

## Abstract

**Background:**

Ischemia-reperfusion acute kidney injury (I/R AKI) is a severe kidney disease with high mortality and morbidity. This study aimed to explore the protective mechanism of glutamine (GLN) against I/R AKI.

**Methods:**

The I/R AKI rat model was established, and HE staining of kidney tissue and serum creatinine (SCr) and blood urea nitrogen (BUN) detection were performed. The miRNAs were sequenced by high throughput in rat kidney tissue samples. Differentially expressed miRNAs (DEmiRs) between the I/R group and I/R + GLN group were screened, and enrichment analysis for target genes of DEmiRs was performed. Meanwhile, human HK-2 cells were cultured, and an I/R model was established to verify the expression of DEmiRs.

**Results:**

Compared with the I/R group, the SCr and BUN levels at each time point were lower in the I/R + GLN group. Vacuolar degeneration of renal tubules in the I/R + GLN group was significantly reduced. In the 104 DEmiRs, we selected miR-132-5p, miR-205, and miR-615 as key miRNAs. KEGG analysis showed that the Notch signaling pathway, PI3K-Akt signaling pathway, and cGMP signaling pathway were mainly related to the GLN against I/R. qRT-PCR verified the downregulation of miR-205 in the I/R group, compared to the sham and I/R + GLN group. The I/R model was established with HK-2 cells, and the expression of miR-132-5p and miR-205 was decreased.

**Conclusion:**

GLN reduced I/R-induced AKI. There were significant differences between miRNAs expression in I/R after GLN treatment. The process of GLN against I/R-induced AKI may be related to the Notch and PI3K-Akt signaling pathway.

## 1. Introduction

Acute kidney injury (AKI) is characterized by acute renal function loss and affects 13.3 million people each year [1]. Among a variety of factors, renal ischemia-reperfusion injury (I/R) is one of the underlying causes of AKI and an inevitable problem in kidney transplantation [2, 3]. I/R-associated AKI is associated with high morbidity and mortality and currently has no effective treatment [4].

In clinical practice, AKI is manifested by accumulation of nitrogen metabolism end products (urea and creatinine) and decreased urine output [5]. In addition, there was concomitant damage to renal tubular epithelial cells and blood vessels, as well as a strong inflammatory response [6, 7]. Recent studies have found that glutamine, a drug used as a conventional nutritional therapy for AKI, could protect the kidney by reducing oxidative stress [8, 9]. Under certain physiological circumstances, glutamine is widely used as a major metabolic fuel for the kidney and immune system [10, 11]. However, its specific protective mechanism is still under study.

microRNAs (miRNAs), a highly conserved small molecule of 21–25 nucleotides, have been reported to be associated with renal I/R and AKI [12, 13]. miRNAs can play a protective role in renal I/R by attenuating the inflammatory response [14]. Although the protective effects of some miRNAs on renal IRI have been discovered, the protective mechanism remains unclear.

So far, there have been few studies on the mechanism of glutamine-mediated kidney protection at the miRNA level. In this study, we applied the high-throughput sequencing technique to analyze the differential expression of miRNAs in renal I/R after glutamine intervention. We further explored the protective mechanism of glutamine against renal I/R at the miRNA level.

## 2. Materials and Methods

### 2.1. Experimental Animal

A total of 72 SPF male Sprague Dawley (SD) rats, weighing 180–260 g and aged 90–120 days, were provided by the Experimental Animal Center of The First Affiliated Hospital of Xinjiang Medical University. Rats were kept in a constant-temperature environment with standardized experimental daily ration without controlling water intake. They were equally randomized into three groups: the sham group, I/R group, and I/R + GLN group. The experimental protocol was approved by the Animal Care and Use Committee in The First Affiliated Hospital of Xinjiang Medical University.

### 2.2. Establishment of the Animal Model

The SD rats were anesthetized by intraperitoneal injection of 2% sodium pentobarbital (40 mg/kg). I/R group: a midline abdominal incision was made and both kidneys were exposed, the right kidney was resected, and the left renal artery was clamped. After 45 minutes, the vascular clamp was removed. The kidney color turned from dark red to bright red, implying that the kinder underwent an I/R pathophysiological process, the rats were observed for 2 hours after closing the abdominal cavity. NS and glutamine (0.75 g/kg) were, respectively, injected by using a micropump from the caudal vein at a rate of 0.5 ml/min after the modeling was completed. Sham group: the right kidney was resected in the same way, and the left renal pedicle was not clamped. Normal saline (NS) was injected by using a micropump from the caudal vein at a rate of 0.5 ml/min after the modeling was completed.

### 2.3. Specimen Collection and Testing

Six rats from each group were randomly selected at each time point (1 h, 5 h, 12 h, and 24 h after modelling), and then, rats were killed under anesthesia. The abdominal aorta was exposed, and venous blood was collected with a syringe. Serum was routinely separated and was stored in a refrigerator at −80°C. The kidneys were quickly resected, and then, one piece of tissue was taken from bilateral kidneys to be placed in 10% neutral formaldehyde and fixed overnight at 4°C.

Serum creatinine (SCr) and blood urea nitrogen (BUN) were tested with a Beckman Automatic Biochemical Analyzer. Paraffin block was prepared from fixed kidney tissue by a process of dehydration, transparency, wax impregnation, and embedding dehydration. Paraffin blocks were then cut into sections, and the staining was performed.

### 2.4. High-Throughput Sequencing Analysis

Total RNA of rat kidney tissue samples from the I/R group and I/R + GLN group was extracted and assessed for quality. According to the small-RNA sequencing library construction process, the purified total renal tissue RNA was reverse-transcribed into cDNA. Then, PCR amplification, purification, and cDNA library quality detection were performed to complete the construction of the sequencing sample library. Then, we obtained the original FASTQ file data.

### 2.5. Bioinformatics Analysis

Clean reads were classified and annotated from the FASTQ file. The Rfam database, species reference transcripts, and repetitive sequence database were used to analyze the known miRNA annotations, miRNA prediction, and miRNA quantification. The differentially expressed miRNAs (DEmiRs) were analyzed using the DESeq R package with |log2FoldChange|>2 and *P* < 0.05. The target gene prediction was performed using RAID and miRanda databases, respectively. Enrichment analysis of Gene Ontology (GO) and KEGG pathways was also performed for target genes using the ClusterProfiler R package. *P* < 0.05 was considered as significant enrichment.

### 2.6. Quantitative Real-Time Polymerase Chain Reaction (qRT-PCR)

Specimens were collected from the rat kidney tissues 24 hours after modeling. Total RNA was extracted, and cDNA products of all miRNAs were obtained using the miRNA first strand cDNA synthesis kit (Shenggong, Shanghai, China). Fluorescence quantitative detection was performed using an miRNA fluorescence quantitative PCR kit (Shenggong, Shanghai, China) with 3 miRNA-specific primers (Table 1). The relative quantitative analysis method (2^−ΔΔCt^) was used to calculate the relative expression of target miRNA in each group of samples. The relative expression of miRNA in each group was calculated using the expression of U6 in each sample in each group as a reference.

### 2.7. Statistical Analysis

The data were analyzed by SPSS19.0. All data were expressed as mean ± standard deviation (mean ± SD). The *t*-test was used for pairwise comparison between groups. *P* < 0.05 indicated that the difference was statistically significant.

## 3. Results

### 3.1. Effect of Glutamine on the Renal Function of Rats after Ischemia Reperfusion

**Figure 1:**
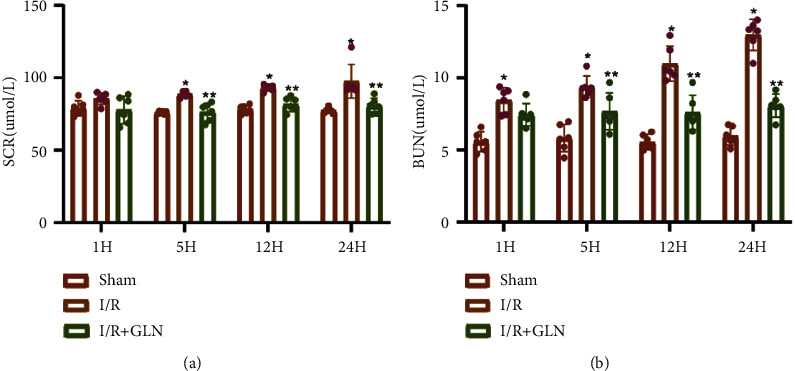
Changes in Scr (a) and BUN (b) levels of rats at different time points in each group. ^*∗*^*P* < 0.05 compared with the sham group; ^#^*P* < 0.05 compared with the I/R group.

The SCr and BUN levels were first examined in the three groups of rats (Figures 1(a) and 1(b)). The SCr and BUN were continued to increase from 1 to 24 hours, reaching a peak at 24 hours in the I/R group, which was significantly higher than those in the sham group (*P* < 0.05). In the I/R + GLN group, SCr and BUN levels were increased from 1 hour to 12 hours, but the increase trend was slower than that in the I/R group. SCr and BUN levels at each time point were lower than those in the I/R group (*P* < 0.05).

### 3.2. Histological Changes in Rat Kidneys

**Figure 2:**
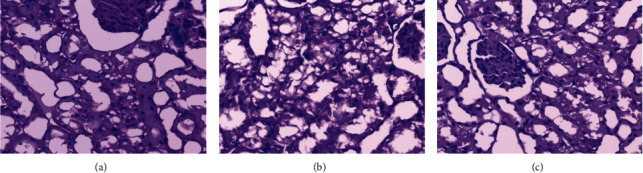
Histopathological changes of rat kidney detected by HE staining. (a) Sham group, (b) I/R group, and (c) I/R + GLN group. Bar: 200×.

In the sham group, after 24 hours of surgery, microscopic observation revealed that some renal tubules in the cortex area were dilated, and some renal tubule epithelia showed edema and vacuolar degeneration, and there was no obvious abnormality in the glomeruli (Figure 2(a)). In the I/R group, the brush borders of renal tubules in the cortical area and the cortex-medullary transitional area disappeared, large numbers of renal tubular epithelial cells showed edema and vacuolar degeneration, while some of them showed karyopyknosis, cytoplasm red stain, coagulation necrosis, abscission, and cast formation (Figure 2(b)). There was no sign of inflammation in the interstitium, and no obvious abnormality was seen in the glomeruli. In the I/R + GLN group, the glomerulus structure was normal under the microscope, some tubular epithelial cells were swollen and showed ballooning degeneration, some tubular epithelial cells were abscised, some tubules were dilated, and a small amount of protein cast was found (Figure 2(c)).

### 3.3. Identification of Differentially Expressed miRNAs

**Figure 3:**
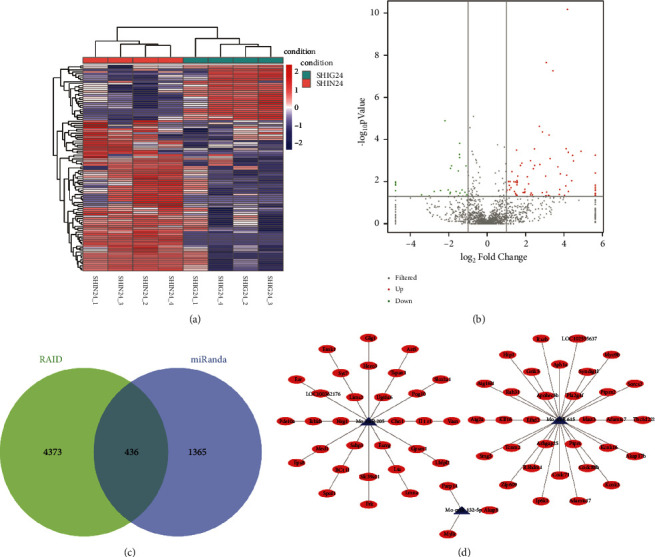
Identification of differentially expressed miRNAs and their regulatory networks. (a) Expression heatmap of differentially expressed miRNAs between the I/R group and I/R + GLN group. Red is upregulation, and blue is downregulation. (b) Volcano plot of differentially expressed miRNAs. Red is upregulation, and green is downregulation. (c) The intersection of predicted target genes in RAID and miRanda. (d) The construction of selected miRNAs-target genes regulator network.

Statistical analysis was performed on the differentially expressed miRNAs screened out from the I/R group and the I/R + GLN group. Compared with the I/R + GLN group, a total of 104 significantly differentially expressed miRNAs were screened out in I/R group (Figure 3(a)). Among them, 76 expressed upregulated miRNA and 28 expressed downregulated miRNA (Figure 3(b)).

**Figure 4:**
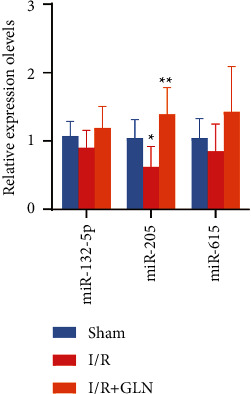
The expression of key miRNAs in the sham group, I/R group, and I/R + GLN group detected by qRT-PCR. ^*∗*^*P* < 0.05 and ^*∗∗*^*P* < 0.01.

In addition, RAID and miRanda were used to perform target gene prediction for the significantly differentially expressed miRNAs. We found 436 intersection target genes (Figure 3(c)). Then, we constructed the regulatory network of miRNA target genes (Figure S1). Importantly, we selected downregulated miR-132-5p, miR-205, and miR-615 in the I/R group as key miRNAs for further study. Also, there were 65 target genes for these three miRNAs (Figure 3(d)). According to the results of qRT-PCR detection in renal tissue, the expression levels of miR-132-5p, miR-205, and miR-615 were decreased in the I/R group (Figure 4).

### 3.4. Biological Functions of Target Genes

**Figure 5:**
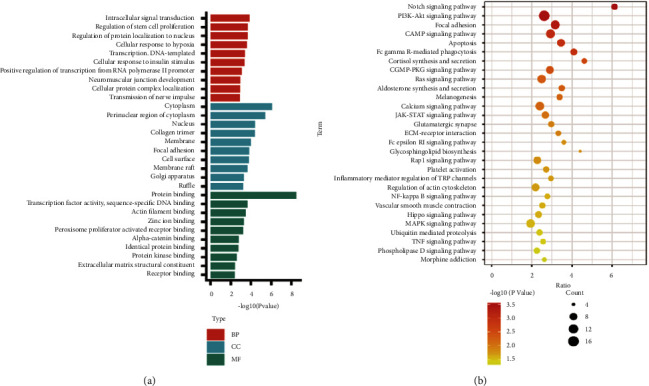
The enrichment analysis of target genes. (a) The top 10 GO terms of target genes enrichment. Red refers to biological processes, blue refers to cell composition, and green refers to molecular function. (b) The KEGG pathways of target genes enriched.

To identify the molecular mechanisms underlying the therapeutic effects of GLN, we performed an enrichment analysis for target genes. In the results of GO (Figure 5(a)), the intracellular signal transduction, regulation of stem cell proliferation, and cellular response to hypoxia of biological processes (BP) were enriched by target genes. Furthermore, the Notch signaling pathway, PI3K-Akt signaling pathway, and cGMP-PKG signaling pathway of KEGG pathways were significantly enriched (Figure 5(b)). Interestingly, rno-miR-132-5p participates in the cGMP-PKG pathway by targeting Mylk.

## 4. Discussion

It's of great clinical value to avoid or reduce renal ischemia-reperfusion injury. GLN has antioxidant and anti-inflammatory effects and can protect the kidney from the severe consequences of ischemia-reperfusion injury [15]. In this study, we found that treatment with GLN significantly decreased SCr and BUN levels and attenuated tubular injury. These effects contributed to its protective effects against I/R-induced AKI. Most importantly, we revealed a novel mechanism of GLN function that was involved in the cGMP-PKG signaling pathway by downregulating miR-132-5p.

miRNAs usually act by binding to the 3′ untranslated regions of target mRNAs, which leads to mRNA degradation and prevents protein translation [16, 17]. In related studies, miRNAs have been found to contribute to the therapeutic target of AKI and kidney repair by regulating inflammation, apoptosis, proliferation, and angiogenesis [18, 19]. Studies on the protective effect of glutamine on the kidney have been carried out in recent years, but there are few studies on the mechanism of glutamine-mediated protection of kidney at the miRNA level.

A total of 104 miRNAs with significant differential expression were screened from the I/R group and I/R + GLN group. Among the key miRNAs we identified, miR-132 plays a great immunoregulatory role in immunity, with CD4 + T cells expressing higher levels of miR-132 and T-cell activation leading to upregulation of miR-132 [20]. miR-132-5p was upregulated in the I/R + GLN group, suggesting that Gln treatment might improve CD4 + T-cell immune activity. miR-205 was significantly downexpressed in AKI patients and was found to be negatively correlated with SCr and BUN and was an independent risk factor for the prognosis of AKI patients [21]. miR-205 is considered a target for the treatment of oxidative stress- and autophagy-related pathological processes during AKI [22, 23]. This implies that GLN may utilize miR-205 to regulate oxidative damage in AKI. miR-615 is downregulated in renal transplantation and focal segmental glomerulosclerosis [24, 25]. However, no studies have been found to show the relationship between miR-615 and I/R.

We further investigated the downstream target regulatory mechanism underlying the altered expression of miRNAs by GLN treatment I/R. In the KEGG pathways, Notch signaling activation plays a promoting role in I/R-associated inflammation and apoptosis [26]. Inhibition of Notch signaling may be a potential anti-inflammatory strategy for the treatment of kidney disease [27]. Activation of the PI3K-Akt signal pathway may play positive roles in antiapoptosis and protecting the kidney from I/R injury [28, 29]. In recent years, the activation of the cGMP/PKG pathway as a therapeutic method to reduce I/R injury has attracted extensive attention, and one of its important protective mechanisms is to participate in the antiapoptotic process [30–32]. These results indicated that GLN may reduce the degree of I/R-induced AKI through the cGMP-PKG pathway.

Some limitations of our study should be taken into account. First, we did not perform high-throughput sequencing of miRNAs expression in the sham group, which may affect the identification of genes involved in GLN treatment. In the cellular model, we did not perform the treatment of GLN to verify the alteration of signaling pathways brought about by its treatment. Future studies will be required to prospectively evaluate key miRNAs as molecular regulatory mechanisms for GLN treatment.

## 5. Conclusions

In conclusion, by means of the high-throughput sequencing technique, this experiment found that, after glutamine intervention, there was a significant differential expression of miRNA in renal histocyte with ischemia-reperfusion injury in vitro, which may be related to the upregulated key miRNAs. The cGMP-PKG pathway influenced by miR-132-5p may be involved in the treatment of I/R by GLN. However, further study is needed to clarify the specific regulatory mechanism.

## Figures and Tables

**Table 1:** Fluorescence quantitative miRNA detection primer information.

Primer	Sequence (5′ to 3′)
rno-miR-132-5p	ACCGTGGCTTTCGATTGTTACT
rno-miR-205	TCCTTCATTCCACCGGAGTCTGT
rno-miR-615	GGGGGTCCCCGGTGCTCGGATC

## Data Availability

The datasets used and/or analyzed during the current study are available from the corresponding author on reasonable request.

## Supplementary Materials

Supplementary Materials Figure S1. The miRNAs-target genes regulator network.
