# Supplementary figures and images for: Analysis of microRNA Expression after Glutamine Intervention in Acute Renal Ischemia-Reperfusion Injury
Source: J Healthc Eng. 2022 Jan 5;2022:2401152. doi: 10.1155/2022/2401152 (PMC8754598; doi:10.1155/2022/2401152)

Figure S1. The miRNAs-target genes regulator network.


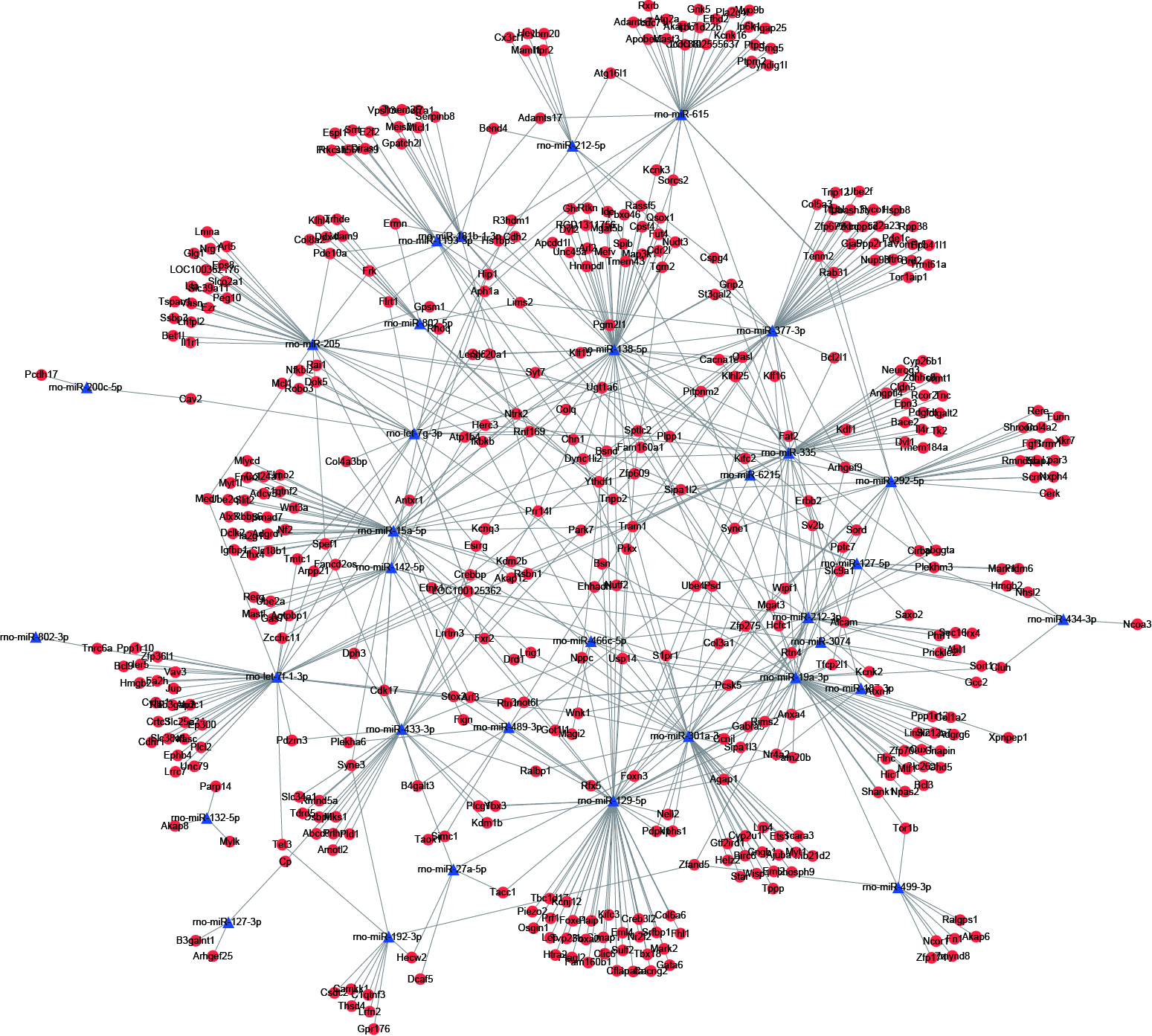

Supplement: Supplementary Materials — Figure S1. The miRNAs-target genes regulator network. [file 2401152.f1.docx]
